# Conotoxin αD-GeXXA utilizes a novel strategy to antagonize nicotinic acetylcholine receptors

**DOI:** 10.1038/srep14261

**Published:** 2015-09-23

**Authors:** Shaoqiong Xu, Tianlong Zhang, Shiva N. Kompella, Mengdi Yan, Aiping Lu, Yanfang Wang, Xiaoxia Shao, Chengwu Chi, David J. Adams, Jianping Ding, Chunguang Wang

**Affiliations:** 1 Institute of Protein Research, Tongji University, 1239 Siping Road, Shanghai 200092, China; 2 National Center for Protein Science Shanghai and State Key Laboratory of Molecular Biology, Institute of Biochemistry and Cell Biology, Shanghai Institutes for Biological Sciences, CAS, 320 Yueyang Road, Shanghai 200031, China; 3 Health Innovations Research Institute, RMIT University, Melbourne, VIC 3083, Australia

## Abstract

Nicotinic acetylcholine receptors (nAChRs) play essential roles in transmitting acetylcholine-mediated neural signals across synapses and neuromuscular junctions, and are also closely linked to various diseases and clinical conditions. Therefore, novel nAChR-specific compounds have great potential for both neuroscience research and clinical applications. Conotoxins, the peptide neurotoxins produced by cone snails, are a rich reservoir of novel ligands that target receptors, ion channels and transporters in the nervous system. From the venom of *Conus generalis*, we identified a novel dimeric nAChR-inhibiting αD-conotoxin GeXXA. By solving the crystal structure and performing structure-guided dissection of this toxin, we demonstrated that the monomeric C-terminal domain of αD-GeXXA, GeXXA-CTD, retains inhibitory activity against the α9α10 nAChR subtype. Furthermore, we identified that His7 of the rat α10 nAChR subunit determines the species preference of αD-GeXXA, and is probably part of the binding site of this toxin. These results together suggest that αD-GeXXA cooperatively binds to two inter-subunit interfaces on the top surface of nAChR, thus allosterically disturbing the opening of the receptor. The novel antagonistic mechanism of αD-GeXXA via a new binding site on nAChRs provides a valuable basis for the rational design of new nAChR-targeting compounds.

Acetylcholine (ACh) is an important neurotransmitter in nervous signal transmission. For the ACh-mediated nerve signals to transmit across synapses or neuromuscular junction (NMJ), ACh is released from the pre-synaptic terminal, and then binds to the extracellular domain of post-synaptic nicotinic acetylcholine receptors (nAChRs), which allosterically leads to the opening of the transmembrane channel of nAChRs to mediate a cationic current^1^. Because of this fundamental role of nAChRs in nerve signal transmission, malfunction of nAChRs is linked to various diseases including myasthenia gravis^2^.

nAChRs are composed of five homologous α- (α1-α10), β- (β1-β4), δ-, ε- or γ-subunits^1^. The muscle-type nAChR has a heteropentameric (α1)_2_β1δγ or (α1)_2_β1δε composition, whereas neuronal nAChRs are either heteropentameric of two α subunits and three auxiliary α or β subunits or a homopentameric α7 subtype. Based on crystal structures of ACh binding proteins (AChBPs) that are structurally homologous to the extracellular domain of nAChRs^3^,^4^, ACh binds to the interface between a principal (+) side of the α subunit and a complementary (−) side of the neighbouring subunit. Thus, the pentameric nAChRs often have two ACh binding sites, the gating mechanism of which is still unknown. Therefore, novel nAChR-specific compounds have great potential for both neuroscience research and clinical applications.

On the other hand, nAChRs are also the targets of various naturally occurring neurotoxins. These nAChR-targeting toxins have not only facilitated structural and functional studies of nAChRs, but also serve as lead compounds in nAChR-targeting drug development^5^. Among those natural toxins, the peptide neurotoxins produced by marine cone snails, generally termed conotoxins, are of particular interest^6^. Several families of conotoxins with different sequences and chemical structures (α-, Ψ-, αB-, αD-, αC-, and αS-conotoxins) can target nAChRs, with α-conotoxins being the most extensively studied ones^7^,^8^,^9^,^10^,^11^. Whilst Ψ-conotoxins are competitive inhibitors of nAChRs via binding to the ACh-binding site^12^,^13^, Ψ- and αD-conotoxins can inhibit nAChRs noncompetitively, at yet unknown binding sites^8^,^14^. In particular, αD-conotoxins occur naturally as a dimer with complex disulfide connections (10 disulfide bonds per dimer)^8^. This makes the structural study of the αD-conotoxin family more challenging and the molecular mechanism of their function more intriguing.

Here we present the crystal structure and electrophysiological activity profile of a dimeric αD-conotoxin GeXXA. Based on these results, we elucidated the mechanism of action of this dimeric conotoxin. Furthermore, we identified the binding site of this conotoxin on nAChRs, which is clearly different from that of ACh. Together, our results establish a new antagonistic mechanism at nAChRs, providing a valuable basis for the rational design of novel nAChR-targeting compounds.

## Results and Discussion

### Identification of αD-conotoxin GeXXA

**Figure 1:**
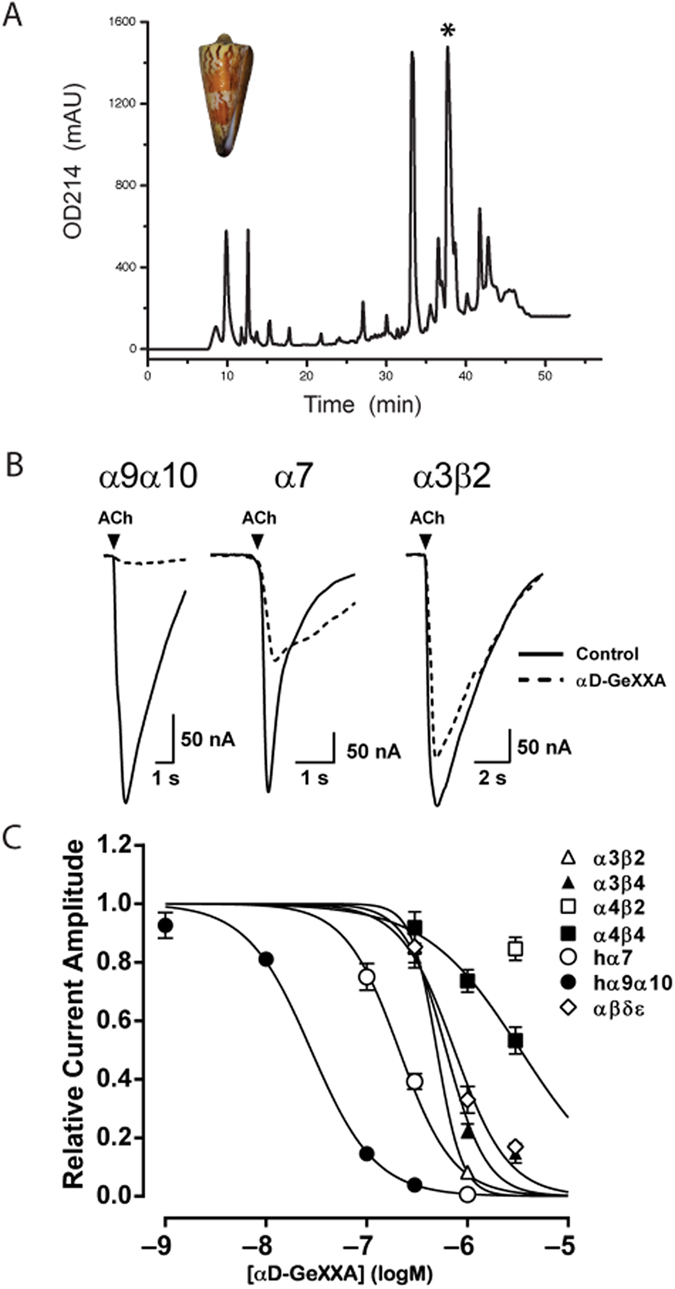
Identification and nAChR-inhibitory activities of αD-GeXXA. (**A**) Separation of a crude venom extraction from *C. generalis* (shown in inset) on a semi-preparative Agilent ZORBAX 300SB-C18 column. The αD-GeXXA peak is indicated with an asterisk. The elution gradient was 5–55% acetonitrile for 0–50 min with a flow rate of 1.5 mL/min. (**B**) Superimposed ACh-evoked current traces in the absence (control) and presence of 300 nM αD-GeXXA (5 min post–incubation). Arrows (▼) indicate ACh application (1 s). (**C**) Concentration-response curves of αD-GeXXA at different nAChR subtypes. Note that, for the comparison between the activities of dimeric and monomeric GeXXA, the concentration of dimeric αD-GeXXA is calculated as the concentration of single subunit.

We identified and isolated a novel αD-conotoxin GeXXA from the venom of *Conus generalis* (Fig 1A). Reduction of this toxin shifted its molecular weight from 11249.0 Da to 5635.0 Da, and alkylation with N-ethylmaleimide (NEM) increased its weight to 6885.0 Da (Fig. S1A). These results indicate this toxin contains 10 Cys residues per peptide and exists as a homodimer with inter-chain disulfide bond(s). N-terminal sequencing and subsequent cDNA cloning of this toxin revealed that each peptide chain of αD-GeXXA comprises 50 amino acid residues sharing high sequence homology with other known αD-conotoxins (Fig. S1).

As αD-conotoxins can act as noncompetitive inhibitors of nAChRs^8^, we first tested the effects of αD-GeXXA on ACh-evoked currents mediated by different nAChR subtypes expressed in *Xenopus* oocytes. Our electrophysiology data showed that αD-GeXXA has strong inhibitory activity on α9α10, α7 and α3β2 subtypes, moderate inhibitory activity on α3β4 and α1β1δε subtypes, and weak activity on α4β2 and α4β4 subtypes (Fig. 1C and Table 1). In particular, αD-GeXXA is most potent against human α9α10 subtype with an IC_50_ of 28 nM.

### Crystal structure of αD-GeXXA

**Figure 2:**
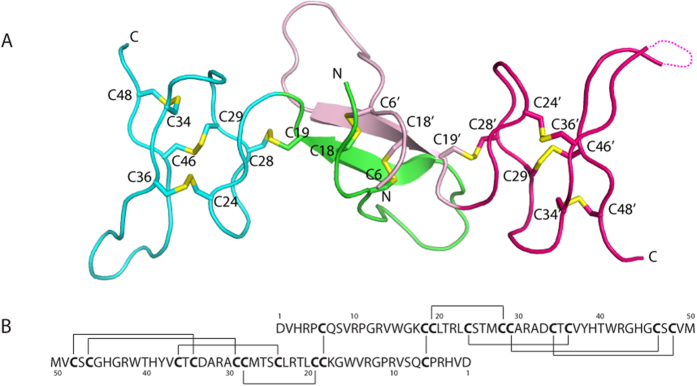
The crystal structure of αD-GeXXA. (**A**) Crystal structure of αD-GeXXA. The NTDs of two monomers are shown in green and pink, and the two CTDs are shown in cyan and magenta, respectively. Disulfide bonds are shown in yellow. (**B**) Sequence and disulfide linkage of αD-GeXXA.

To gain insight into its biological function, we determined the crystal structure of native αD-GeXXA using *ab initio* methods^15^ and refined it to 1.5 Å resolution (Table S1 and Fig. 2). There is one conotoxin homodimer with a pseudo two-fold symmetry per asymmetric unit. Each peptide chain consists of an N-terminal domain (NTD, residues 1–20) and a C-terminal domain (CTD, residues 21–50). The NTD comprises an N-terminal loop and a β-strand, and the CTD assumes several extended loop conformations. The dimerization involves mainly the N-terminal loops and β-strands of the NTDs, which is further stabilized by two inter-chain disulfide bonds between Cys6 of one chain and Cys18 of the other. There are three disulfide bonds (Cys24-Cys36, Cys29-Cys46 and Cys34-Cys48) in the CTD, making the CTD adopt a compact structure. The two CTDs flank the dimeric NTDs, and the relative conformation of the NTD and the CTD is stabilized by a disulfide bond between Cys19 and Cys28 (Fig. 2).

### Preparation of monomeric GeXXA-CTD

Interestingly, the CTD of αD-GeXXA adopts a canonical inhibitory cystine knot (ICK) disulfide linkage, as observed in many 6-Cys-residue-containing bioactive peptides, including several families of conotoxins^16^. This observation prompted us to speculate that the CTD of αD-GeXXA alone may exhibit inhibitory activity against nAChRs. To obtain an isolated CTD, we first synthesized a peptide of residues 21–50, with Cys28 replaced by Ser and the thiol groups of both Cys24 and Cys36 protected by the acetamidomethyl groups (Acm) (Fig. S2A). Oxidation of the synthetic peptide with GSH/GSSH yielded two major products (Fig. S2B). Partial reduction and LC-MS/MS analysis showed that the product in peak 1 has the correctly connected Cys29-Cys46 and Cys34-Cys48 disulfide bonds (Fig. S3). Subsequent iodine oxidation of this intermediate product led to formation of the third disulfide bond between Cys24 and Cys36, thus yielding the monomeric CTD (Fig. S2C).

### GeXXA-CTD has nAChR-inhibitory activity

**Figure 3:**
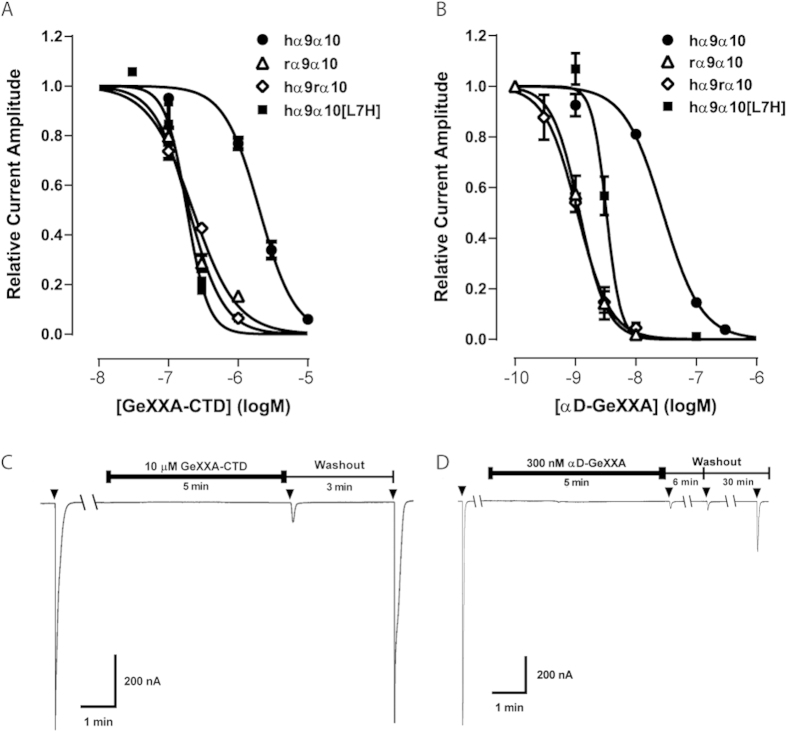
The nAChR-inhibitory activities and different dissociation kinetics of monomeric GeXXA-CTD and dimeric αD-GeXXA. (**A**) Concentration-response curves of GeXXA-CTD at human α9α10 (●), rat α9α10 (△), hybrid hα9rα10 (◇) and human α9α10[L7H] (◼) receptors. (**B**) Concentration-response curves of αD-GeXXA at human α9α10 (●), rat α9α10 (△), hybrid hα9rα10 (◇) and human α9α10[L7H] (◼). (**C,D**) GeXXA-CTD (10 μM) (Panel **C**) has faster washout kinetics when compared to αD-GeXXA (300 nM) (Panel D) at hα9α10 nAChR expressed in *Xenopus* oocytes. Arrows (▼)indicate ACh application (1 s).

Indeed, the monomeric GeXXA-CTD showed inhibitory activity against human α9α10 subtype (IC_50_ of 2.02 μM), but had little or no effect on other nAChR subtypes (Table 1). In general, the activity of GeXXA-CTD is weaker than that of the full-length dimeric αD-GeXXA, making GeXXA-CTD apparently specific to the α9α10 subtype. While focusing on this subtype, we found that GeXXA-CTD has a 10-fold higher potency on rat α9α10 (IC_50_ of 198 nM) than on human α9α10 nAChR (Fig. 3A and Table 2). Interestingly, GeXXA-CTD also exhibited a comparably high potency (IC_50_ of 224 nM) on a hybrid receptor of human α9 and rat α10 subunits (hα9rα10)^17^ (Fig. 3A). These results suggest that the α10 subunit determines the preference of GeXXA-CTD for rat over human α9α10 nAChR. Since the nAChR-inhibitory activities of αD-GeXXA and GeXXA-CTD were measured after extracellular application (see materials and methods), the species preference might be due to residue differences in the extracellular domain of human and rat α10 subunits.

### Identification of the binding site of αD-GeXXA

To investigate the potential binding site of the α9α10 nAChR for αD-GeXXA, we compared the sequences of the extracellular domains of human and rat α10 subunits and identified differences in 12 residues (Fig. S4). Based on the EM structure of *Torpedo* α1β1δγ nAChR^18^ and the crystal structures of AChBP^12^, the corresponding positions of these 12 differing residues are mostly well scattered on the surface of the extracellular domain (Fig. S5A and B). However, the pseudo two-fold symmetry of the two CTDs in the dimeric αD-GeXXA implies existence of two equivalent binding sites on each nAChR, presumably on two non-adjacent subunits. On the other hand, the length of αD-GeXXA (up to 52.4 Å between the Cα atoms of the two C-terminal Met50 residues) makes it unlikely that αD-GeXXA binds in the nAChR central pore or on the outside-facing surface of the pentameric extracellular domains (Fig. S5C). Thus, the top surface of nAChR seems the most likely binding site for αD-GeXXA. Among the 12 differing residues, only residue 7 is located on the top surface of nAChR. We therefore hypothesized that His7 is the likely candidate conferring specificity of αD-GeXXA to rat α10 subunit.

To verify the functional role of residue 7 of nAChR α10 subunit in αD-GeXXA binding, we mutated Leu7 of human α10 subunit to His, the corresponding residue in rat α10 subunit. The inhibitory activity of a competitive α-conotoxin Vc1.1 was not affected by this mutation (data not shown), thus excluding the possibility of the mutation introducing significant structural change. Remarkably, GeXXA-CTD showed an IC_50_ of 183 nM on this hα9α10[L7H] mutant. This is comparable to the activity on rα9α10 but is 11-fold lower than that on hα9α10 (Fig. 3A and Table 2), strongly suggesting that His7 of rat α10 subunit is involved in the interaction with GeXXA-CTD.

To further confirm this potential binding site for αD-conotoxins, the activity of the natural dimeric αD-GeXXA was also measured on these nAChR subtypes. The dimeric αD-GeXXA exhibited a strong preference on rat α9α10 subtype rather than human α9α10 subtype, the IC_50_ on rat α9α10 subtype (1.2 nM, Fig. 3B and Table 2) being clearly lower than that on human α9α10 subtype. Similar to the situation of GeXXA-CTD, the dimeric αD-GeXXA showed the same potency on the hybrid receptor of human α9 and rat α10 subunits (hα9rα10), with an IC_50_ of 1.2 nM (Fig. 3B and Table 2). Furthermore, the L7H mutation of human α10 subtype clearly enhanced the potency of αD-GeXXA, its IC_50_ on hα9α10[L7H] getting close to that on rα9α10 and hα9rα10 (Fig. 3B and Table 2). These results further support the notion that His7 of the rat α10 subunit confers the species preference of αD-GeXXA and may serve as the binding site for αD-GeXXA.

### A cooperative two-site binding model of αD-GeXXA

**Figure 4:**
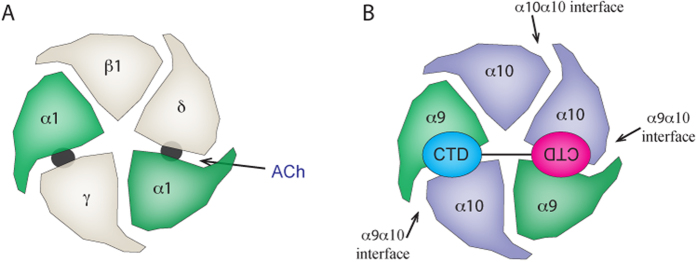
Binding model of αD-GeXXA on nAChR. (**A**) The well-established binding site stoichiometry of ACh on the muscle nAChR subtype. (**B**) Binding model of αD-GeXXA on the α9α10 subtype of nAChR. The two CTDs of αD-GeXXA, which are shown in cyan and pink, respectively, with roughly 180° rotation, bind at the top surface of the two “α9α10” interfaces to inhibit the opening of the nAChR.

Based on sequence alignment, the critical residue, His7 of rat α10 subunit, corresponds to Asn9 of the *Torpedo* δ subunit or Glu8 of the *Torpedo* γ subunit (Fig. S4), which is located on the complementary (−) side of each subunit and faces towards the principal (+) side of its clockwise adjacent subunit (Fig. S5C). Therefore, the binding site of αD-GeXXA is probably located at the interface between the α10 subunit and its clockwise adjacent subunit. In the pentameric α9α10 nAChR, there are two α9 and three α10 subunits^19^. Following the nomenclature for ACh-binding sites on nAChR^4^, the three α10-involving interfaces would be two “α9α10” interfaces and one “α10α10” interface, all of which could be potential binding sites for αD-GeXXA. However, the pseudo two-fold symmetry and the length of the dimeric full-length αD-GeXXA suggest that the two CTDs of this toxin most likely bind the two “α9α10” interfaces at the top surface of α9α10 nAChR (Fig 4). By doing so, αD-GeXXA, and possibly all the αD-conotoxins, can allosterically and cooperatively perturb the conformational changes of the receptor and opening of the channel.

This cooperative, two-site binding model of αD-GeXXA is also supported by our electrophysiological data. Firstly, inhibition of nAChR by both αD-GeXXA and GeXXA-CTD often gave Hill slopes greater than 1 (Tables 1 and 2), suggesting cooperative rather than single-site binding on nAChR. Secondly, the dimeric αD-GeXXA is considerably more potent than the monomeric GeXXA-CTD (Table 1 and Fig. 3). Thirdly, the dimeric αD-GeXXA exhibits much slower dissociation kinetics than the monomeric GeXXA-CTD (Fig. 3C,D). Interestingly, all these properties have been observed in a study of polymer-linked ligand dimers^20^. We now show that this dimerization strategy is adopted by a natural toxin to gain higher potency.

In summary, by resolving the crystal structure and performing structure-guided dissection of a dimeric conotoxin αD-GeXXA, we demonstrate that αD-conotoxins inhibit nAChR most likely by binding of the two CTDs cooperatively to two inter-subunit interfaces on the top surface of nAChR. This working mechanism is distinct from that of another dimeric conotoxin that targets AMPA receptors^21^. The binding site of αD-conotoxin on nAChR differs from the binding sites of the endogenous ligand ACh, and the competitive α-conotoxins^12^,^13^ and α-bungarotoxin^22^. However, the binding site stoichiometry of αD-GeXXA is coincidently the same as that of ACh on the muscle subtype and neuronal heterogeneous nAChR (that is, 2 of 5 inter-subunit interfaces)^23^ (Fig. 4). The cooperative inhibitory mechanism of αD-GeXXA via a novel binding site on nAChRs provides a valuable basis for the rational design of new nAChR-targeting drugs.

## Methods

### Toxin purification and characterization

*Conus generalis* specimens were collected from the South China Sea. To extract the crude venom, the venom duct of living snails was dissected into short fragments and venom was extracted successively with 0.1% (v/v) trifluoroacetic acid (TFA), and 0.1% TFA in 20%, 30%, 40% and 50% acetonitrile. Supernatants were pooled and lyophilized.

For toxin purification, the lyophilized crude venom was dissolved in 0.1% TFA, and the soluble supernatant was separated on a Zobax C18 column (250 × 4.6 mm, Agilent) with an acetonitrile gradient using an Agilent 1100 HPLC system. Reduction of the purified αD-GeXXA was carried out in 100 mM Tris-HCl, pH 8.7, 2 mM EDTA and a 100-fold excess of dithiothreitol (DTT) at 37 °C for 1 h. Alkylation of the reduced thiol groups of αD-GeXXA was carried out in the same buffer containing 10 mM N-ethylmaleimide (NEM) at 37 °C in the dark for 0.5 h. After being purified on a C18 HPLC column (Fig. S1), the reduced and alkylated αD-GeXXA was applied to an ABI 491A Procise Protein Sequencing System for N-terminal sequencing. The N-terminal partial sequence DVHRPCQSVRPGRVWGKCCLT was obtained.

### cDNA cloning

Total RNA was extracted from homogenized venom ducts of *Conus generalis* with TRIZOL reagent, according to the manufacturer’s protocol. The 3′-partial cDNA of αD-GeXXA was cloned using a 3′-RACE kit (Invitrogen) and a gene specific primer 1 (5′-GAYGTNCAYCGNCCNTGYCAR-3′, Y: T/C, R: G/A, N: A/T/G/C), encoding αD-GeXXA N-terminal sequence DVHRPCQ. The 5′-partial cDNA of αD-GeXXA was cloned using a 5′-RACE kit (Takara) with gene specific primers (GSP2: 5′-GATTGCACTCAGGCAGATCA-3′; GSP3: 5′- CGGTTGCTCTTTGAT TGGTT-3′; GSP4: 5′-CATTACGCAGGAACACCCGTG-3′) based on the 3′-partial cDNA sequence. Overlapping of the 3′- and 5′-partial cDNA sequences gave the full-length cDNA of αD-GeXXA (Fig. S1D).

### Electrophysiological recordings from nAChRs exogenously expressed in *Xenopus* oocytes

RNA preparation, oocyte preparation, and expression of nAChR subunits in *Xenopus* oocytes were performed as described previously^24^. Briefly, plasmids with cDNAs encoding the rat α1, α3, α4, α9, α10, β1, β2, β4, δ, ε and human α7 subunits subcloned into the oocyte expression vector pNKS2 and human α9 and α10 subunits subcloned into the pT7TS vector were used for mRNA preparation using the mMESSAGE mMACHINE Kit (Ambion Inc., USA). All oocytes were injected with 5 ng of cRNA and kept at 18 °C in ND96 buffer (96 mM NaCl, 2 mM KCl, 1 mM CaCl_2_, 1 mM MgCl_2_, and 5 mM HEPES, pH 7.4), supplemented with 50 mg/L gentamycin and 100 μg/ml penicillin/streptomycin for 2–5 days before recording. Membrane currents were recorded from *Xenopus* oocytes using a GeneClamp 500B amplifier (Molecular Devices) in a two-electrode (virtual ground circuit) voltage-clamp setup. Both the voltage-recording and current-injecting electrodes were pulled from borosilicate glass (GC150T - 7.5, Harvard Apparatus Ltd.) and had resistances of 0.3–1.5 MΩ when filled with 3 M KCl. All recordings were conducted at room temperature (21–23 °C) using a bath solution of ND96 as described above. During recording, the oocytes were perfused continuously at a rate of 1.5 ml/min, with 300 s incubation times for peptides. Acetylcholine (200 μM for α7 and 50 μM for all other nAChR subtypes) was applied for 1 s at 2 ml/min, with 3–4 min washout periods between applications. Cells were voltage-clamped at a holding potential of −80 mV. Data were filtered at 100 Hz and sampled at 500 Hz. Peak ACh-evoked current amplitude was measured before and after incubation with peptide.

Concentration-response curves for antagonists were fitted by unweighted nonlinear regression to the following logistic equation 1


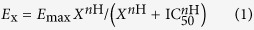


where *E*_x_ is the response, *X* is the antagonist concentration, *E*_max_ is the maximal response, *n*^H^ is the slope factor, and IC_50_ is the antagonist concentration giving 50% inhibition of maximal response. All electrophysiological data were pooled (*n* = 4–8 oocytes for each data point) and represent arithmetic means ± standard error of the fit. Computation was done using GraphPad Prism 6 (GraphPad Software Inc., La Jolla, CA, USA).

### Crystallization and structure determination

The powder of 1.0 mg native αD-GeXXA was dissolved in a buffer containing 20 mM Tris-HCl (pH 8.0) and 100 mM NaCl. Crystallization of αD-GeXXA was performed using the hanging drop vapor diffusion method by mixing 1.5 μl protein solution (about 10 mg/ml) and 1.5 μl reservoir solution at 16 °C. Crystals were grown from drops consisting of a reservoir solution of 0.1 M citrate acid (pH 5.0) and 10% PEG 6000 after about 1 week. The diffraction data were processed, integrated, and scaled together as two datasets with HKL2000^25^. Dataset 1 was processed at high resolution for *ab initio* phasing and dataset 2 with reasonable statistics was used for structure refinement.

The structure of αD-GeXXA was determined by *ab initio* methods using the program Acorn^15^. Phases were determined with dataset 1 by setting optimal Acorn parameters to start from a random atom (no prior knowledge) to determine substructure and then applied to the program Acorn-MR to produce an interpretable electron density map. An initial model of 63 out of 100 residues for two monomers was constructed automatically by warpNtrace mode of ARP/wARP^26^. The remaining residues and additional water molecules were built manually using Coot with dataset 2 27,28. Structure refinement was performed using Refmac5 and Phenix^27^,^29^. The stereochemistry of the protein model was analyzed using MolProbity^30^. Structure analysis was carried out using programs in CCP4^31^. Figures were generated using Pymol (http://www.pymol.org). Statistics of the structure refinement and the quality of the final structure model are summarized in Table S1.

### Preparation of GeXXA-CTD

The linear peptide of GeXXA-CTD, with Cys24 and Cys36 protected by Acm, was synthesized by the Chinese Peptide Company (Hangzhou, China). The peptide was first oxidized with 1 mM GSSG/GSH (Fig. S2), which produced two products. To examine the disulfide linkage, the first peak (Peak 1) was partially reduced with Tris(2-carboxyethyl)phosphine (TCEP) and alkylated with NEM, and then fully reduced with DTT and alkylated with iodoacetamide (IAA). The resultant product of Peak 1 was digested with trypsin and analyzed with LC-MS/MS on an Orbitrap Elite (ThermoFisher, USA), revealing the Cys29-Cys46 and Cys34-Cys48 linkages (Fig. S3). Peak 1 was then treated with iodine to remove the Acm group and oxidize the disulfide bond between Cys24 and Cys36 (Fig. S2).

## Additional Information

**Accession codes:** The cDNA sequence of αD-GeXXA has been deposited in the GenBank database with accession number (KM373785). The atomic coordinates and structure factors of αD-GeXXA have been deposited in the Protein Data Bank (http://www.wwpdb.org/) with accession code 4X9Z.

**How to cite this article**: Xu, S. *et al*. Conotoxin αD-GeXXA utilizes a novel strategy to antagonize nicotinic acetylcholine receptors. *Sci. Rep*. **5**, 14261; doi: 10.1038/srep14261 (2015).

## Supplementary Material

Supplementary Information

## Figures and Tables

**Table 1:** Inhibition of different nAChR subtypes by dimeric αD-GeXXA and monomeric GeXXA-CTD.

nAChRsubtype	αD-GeXXA		GeXXA-CTD	
	IC_50_ (95% CI)	Hill Slope (n^H^)	IC_50_ (95% CI)	Hill Slope (n^H^)
hα9α10	28 nM (22–35)	−1.3	2.02 μM (1.82–2.25)	−1.7
hα7	210 nM (174–253)	−2.2	−a	−
rα3β2	498 nM (407–609)	−3.5	−	−
rα3β4	614 nM (491–768)	−1.6	−	−
rα4β2	>3 μM	−	−	−
rα4β4	>3 μM	−0.9	−	−
rα1β1δε	743 nM (606–911)	−1.6	−	−

^a^undetectable.

**Table 2:** Inhibitory activities of monomeric GeXXA-CTD and dimeric αD-GeXXA on α9α10 nAChR from different species.

α9α10 nAChR	GeXXA-CTD		αD-GeXXA	
	IC_50_ (95% CI)	Hill Slope (n^H^)	IC_50_ (95% CI)	Hill Slope (n^H^)
hα9α10	2.02 μM (1.82–2.25)	−1.7	28 nM (22–35)	−1.3
rα9α10	198 nM (164–238)	−1.7	1.2 nM (1.0–1.4)	−1.9
hα9rα10	224 nM (194–258)	−1.4	1.1 nM (0.9–1.3)	−1.6
hα9α10[L7H]	183 nM (132–255)	−2.8	3.3 nM (2.8–3.8)	−3.6
