# Supplementary material for: Conotoxin αD-GeXXA utilizes a novel strategy to antagonize nicotinic acetylcholine receptors
Source: Sci Rep. 2015 Sep 23;5:14261. doi: 10.1038/srep14261 (PMC4585798; doi:10.1038/srep14261)
Supplement: Supplementary Information [file srep14261-s1.doc]

Conotoxin αD-GeXXA utilizes a novel strategy to antagonize nicotinic acetylcholine receptors

Shaoqiong Xu^†,#^, Tianlong Zhang^‡,#^, Shiva N. Kompella^⊥,#^, Mengdi Yan^†^,

Aiping Lu^†^, Yanfang Wang^†^, Xiaoxia Shao^†^, Chengwu Chi^†,‡^,

David J. Adams^⊥*^, Jianping Ding^‡*^, and Chunguang Wang^†*^

^†^ Institute of Protein Research, Tongji University, 1239 Siping Road, Shanghai 200092, China;

^‡^ National Center for Protein Science Shanghai and State Key Laboratory of Molecular Biology, Institute of Biochemistry and Cell Biology, Shanghai Institutes for Biological Sciences, CAS, 320 Yueyang Road, Shanghai 200031, China;

^⊥^ Health Innovations Research Institute, RMIT University, Melbourne, VIC 3083, Australia.

^#^ These authors contributed equally to this work.

*Correspondence author: [david.adams@rmit.edu.au](mailto:david.adams@rmit.edu.au) or [jpding@sibcb.ac.cn](mailto:jpding@sibcb.ac.cn) or chunguangwang@tongji.edu.cn

**Supplementary Table S1;**

**Supplementary Figures S1-S5.**

**Supplementary Table S1. Summary of diffraction data and structure refinement statistics**

**Summary of diffraction data 1 2**

Wavelength (Å) 1.0000 1.0000

Space group *P*2_1_2_1_2_1_ *P*2_1_2_1_2_1_

Cell parameters

a (Å) 25.4 25.4

b (Å) 42.4 42.4

c (Å) 83.0 83.0

Resolution range ^a^ (Å) 50.0-1.20 50.0-1.50

(1.24-1.20) (1.55-1.50)

Observed reflections 155,090 88,707

Unique reflections (I/σ(I)>0) 26,222 13,989

Average redundancy 5.9 (4.2) 6.3 (6.3)

Average I/σ(I) 33.9 (0.35) 47.5 (4.6)

Completeness (%) 90.4 (69.7) 92.8 (94.4)

R_merge_ (%) 3.9 (0.0) 3.6 (41.8)

**Statistics of refinement and model**

Number of reflections (F_o_>0σ(F_o_))

Working set 12,527

Free R set 709

R factor / Free R factor (%) 17.9 / 22.3

Number of protein atoms 731

Number of water atoms 69

Average B factor of all atoms (Å^2^)

All atoms 41.3

Protein main chain / side chain 36.8 / 44.9

Water 47.6

RMS bond lengths (Å) 0.008

RMS bond angles (°) 1.3

Ramachandran plot (%)^b^

Favored 93.3

Allowed 100

^a^ Numbers in parentheses refer to the highest resolution shell.

^b^ Statistics of the Ramachandran plot was analyzed using MolProbity.


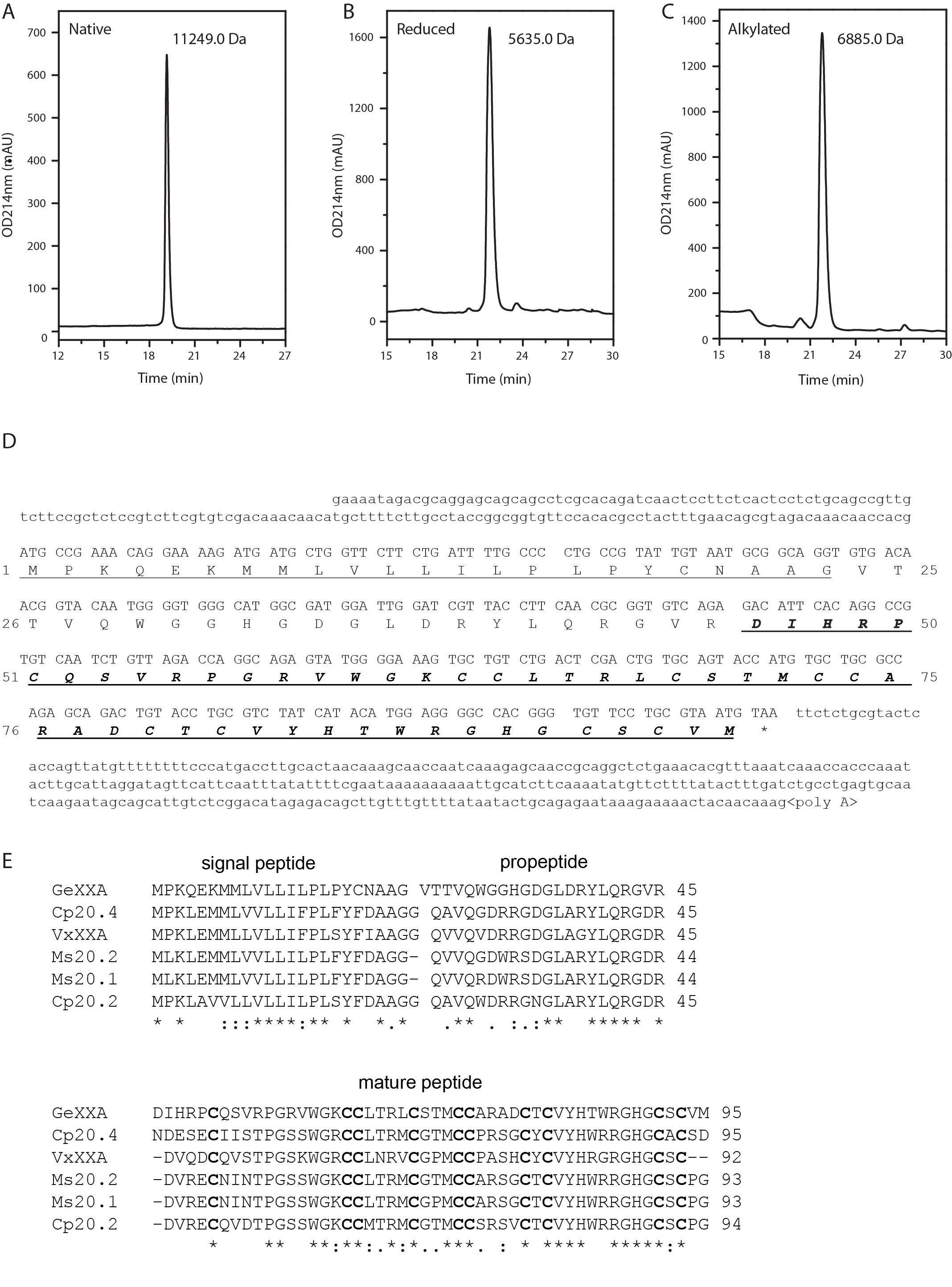


**Fig. S1: The characterization and cDNA cloning of αD-GeXXA.** (A) The HPLC profile of purified native αD-GeXXA. (B) The HPLC profile of reduced αD-GeXXA. (C) The HPLC profile of reduced and alkylated αD-GeXXA. The purification of (A), (B) and (C) were performed with an analytic Agilent ZOBAX C18 column and a 0.1% TFA/acetonitrile system with an elution gradient of 45-60% acetonitrile in 10-40 min. (D) The cDNA sequence of αD-GeXXA and the cDNA-encoded precursor sequence. The coding region of cDNA is shown in capital letters. The signal peptide sequence is underlined, and the mature peptide sequence is shown in bold italic and underlined. The second residue of the mature peptide (Ile) is different from the residue at the corresponding position (Val) obtained from Edman-degradation sequencing, which is probably due to the polymorphism of conotoxins. (E) Sequence alignment of the precursors of αD-GeXXA and other αD-conotoxins.


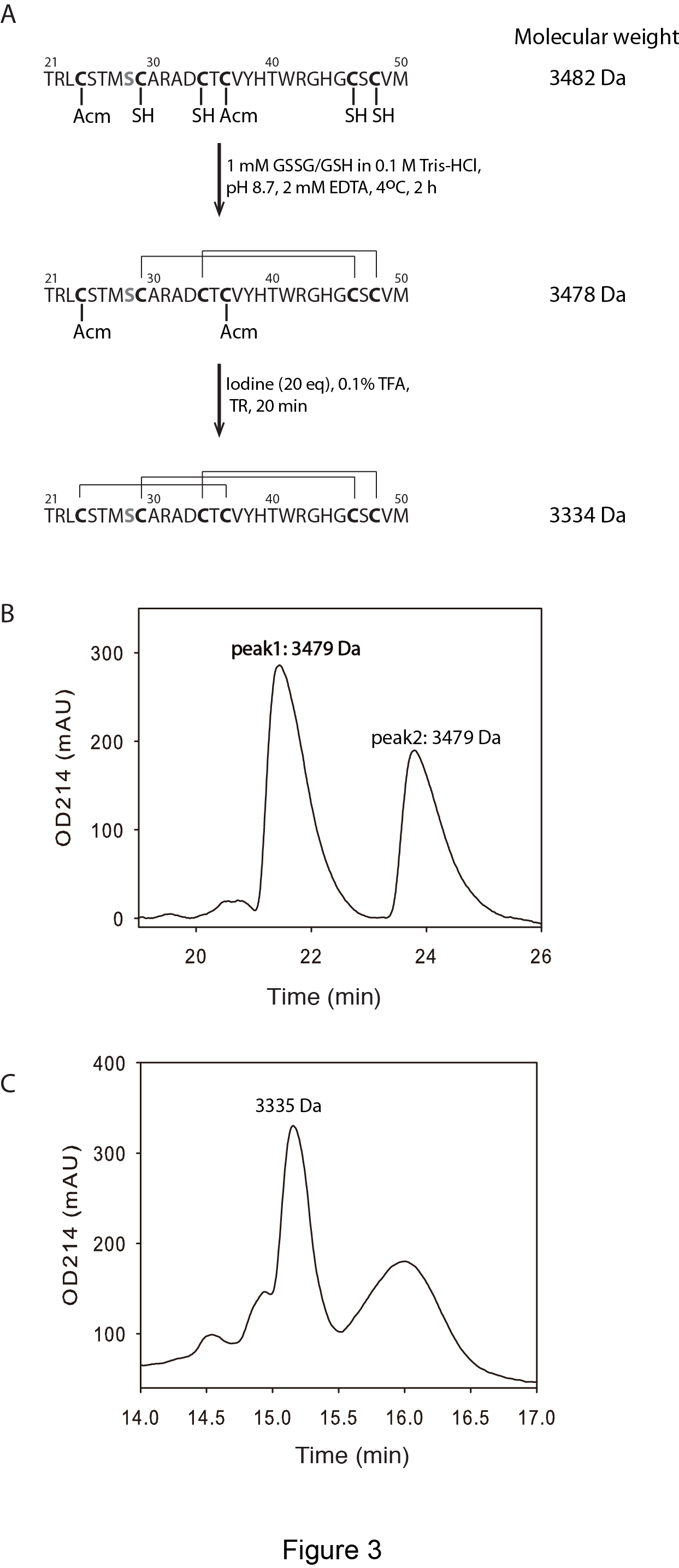


**Fig. S2. Total synthesis of monomeric GeXXA-CTD.** (A) Procedure and conditions for the preparation of correctly folded CTD. (B) The GSSG/GSH oxidation product of the linear peptide of CTD was separated on an analytical HPLC C18 column by isocratic elution with 22% acetonitrile in 0.1% TFA. The first major peak was the product with expected disulfide connection (see Fig. S3). (C) The final iodine oxidation product of the CTD was separated on an analytical Agilent HPLC C18 column with 25-40% acetonitrile gradient for 15 min. The measured molecular weight of each product is shown with each peak.


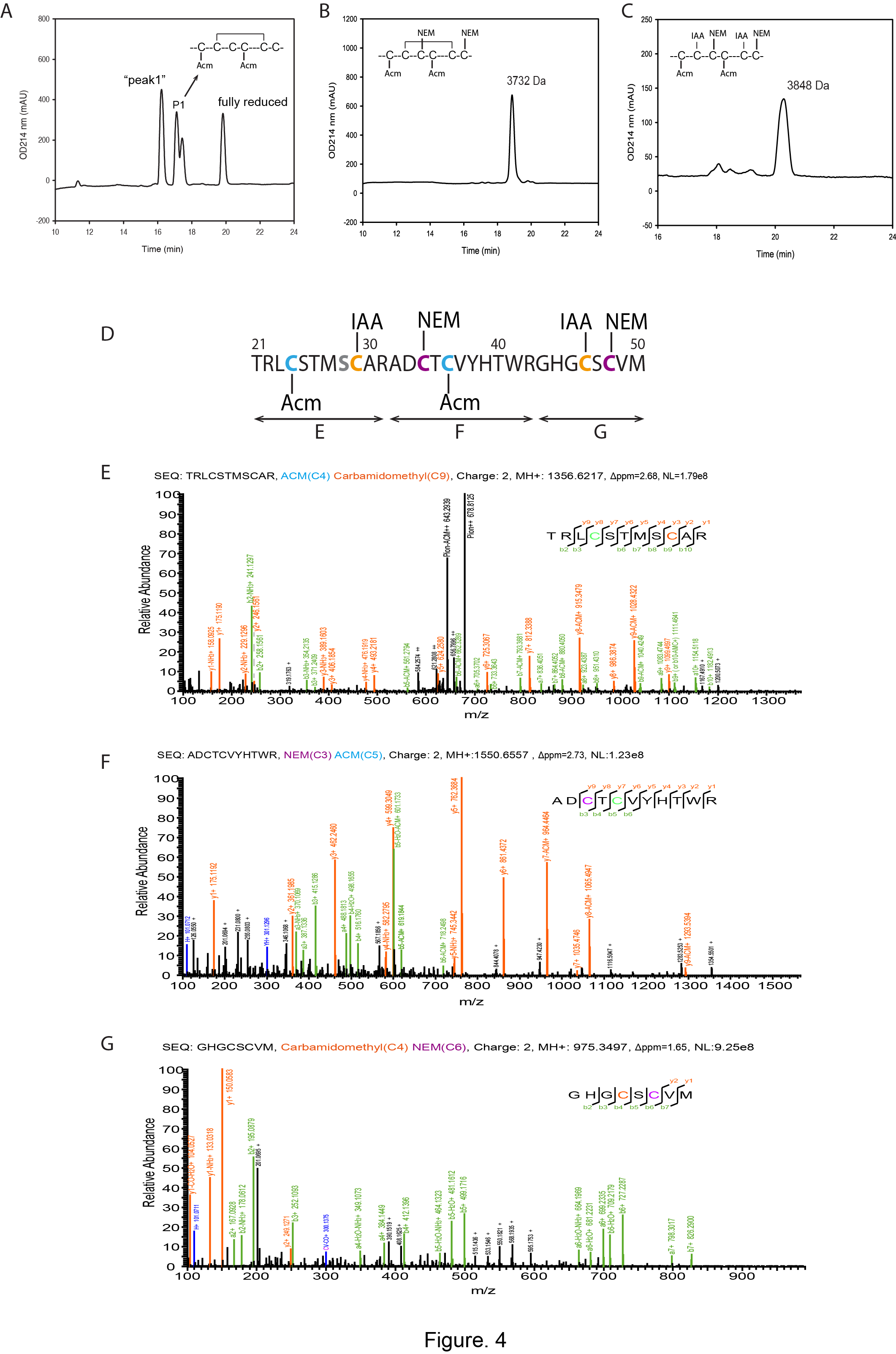


**Fig. S3**: **Identification of the disulfide bond linkage of Peak1 of GSSG/GSH oxidation product of linear CTD in Fig. S2B.** (A) Product of TCEP partial reduction of Peak1. P1 was used for further alkylation and reduction. (B) Product of N-ethylmaleimide (NEM) alkylation of P1. (C) Product of full reduction and iodoacetamide (IAA) alkylation of NEM-modified P1. The HPLC elution of Panels A, B and C was performed on a C18 column with a 25-40% acetonitrile gradient for 15 min. (D) The final product of stepwise reduction and alkylation of peak1. Trypsin digestion of the final product gave three fragments whose MS/MS spectra are shown in Panels E, F and G, respectively.


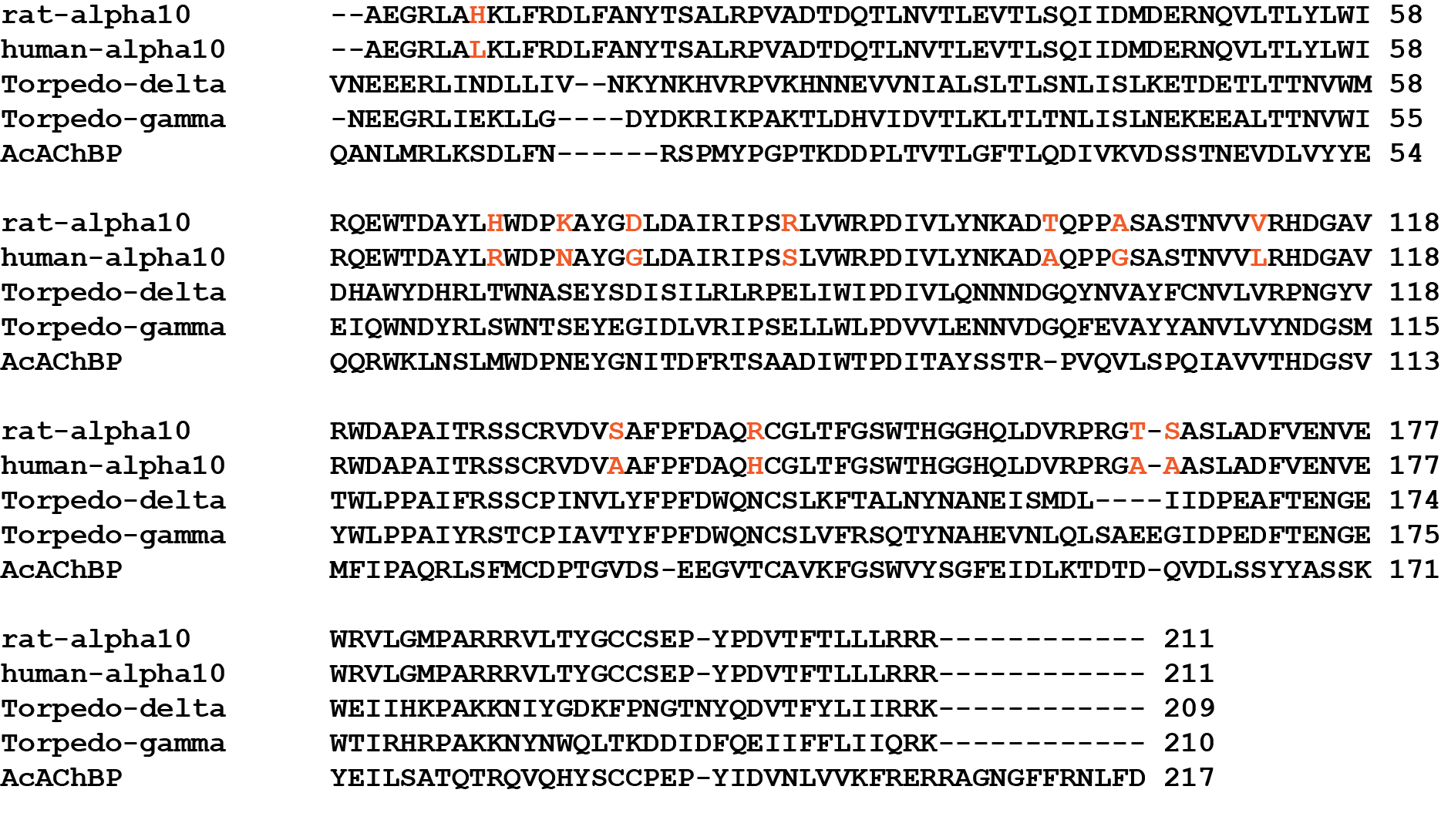


**Fig. S4: Sequence alignment of the extracellular domains of rat α10, human α10, Torpedo δ and Torpedo γ subunits of nAChR, and AcAChBP.** The sequences were aligned by ClustalW2 with default parameters (http://www.ebi.ac.uk/Tools/msa/clustalw2/). Residues non-identical between rat α10 and human α10 extracellular domains are indicated in orange.


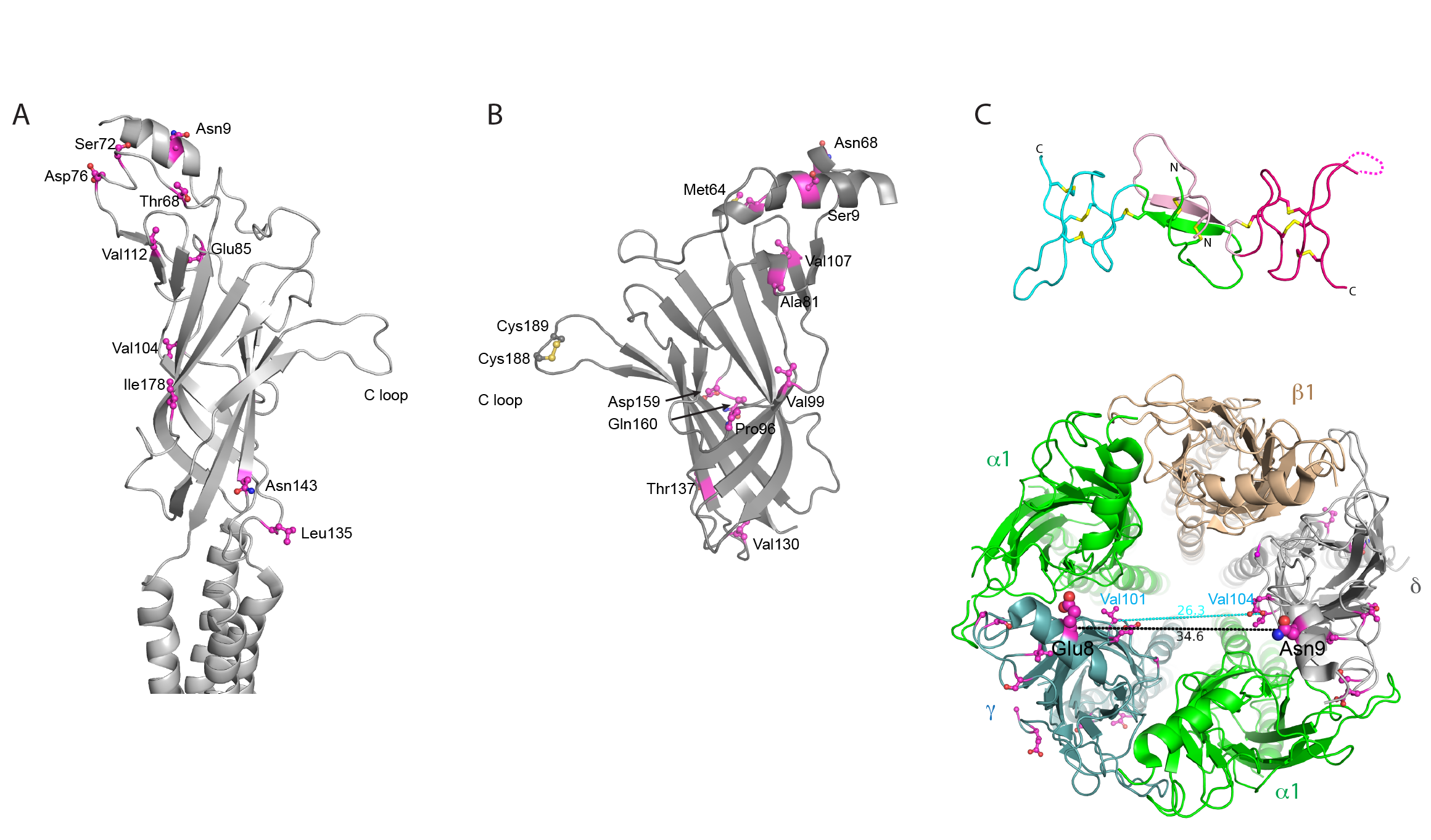


**Fig. S5: The binding site of αD-GeXXA on nAChR.** (A) and (B) Positions of the residues corresponding to those differing between the extracellular domains of rat and human α10 subunits are mapped in the *Torpedo* nAChR δ subunit (PDB 2BG9) (A) and AcAChBP (PDB 2BR8) (B). The C loop is indicated to show that Torpedo nAChR δ subunit and AcAChBP are presented in different orientation. (C) Top view of αD-GeXXA (upper panel) and Torpedo nAChR (lower panel). αD-GeXXA and Torpedo nAChR are shown in the same scale, highlighting that the two CTDs of αD-GeXXA have the appropriate distance and orientation to bind to two nonadjacent interfaces on the top surface of nAChR. In *Torpedo* δ and γ subunits, the residues corresponding to those differing between the extracellular domains of rat and human α10 subunits are shown in magenta and in stick-and-ball mode. Asn9 of the *Torpedo* δ subunit and Glu8 of the *Torpedo* γ subunit correspond to His7 of the rat α10 subunit, and are highlighted with larger sphere. The distance between their Cα atoms, 34.6 Å, is comparable to the size of αD-GeXXA, given it is located on the top surface of nAChR. In contrast, the distance between Cα atoms of another pair of variant residues in the central pore surface (Val104 of δ subunit and Val101 of the γ subunit), 26.3 Å, is too short to accommodate the αD-GeXXA molecule.
